# Austria’s Digital Vaccination Registry: Stakeholder Views and Implications for Governance

**DOI:** 10.3390/vaccines9121495

**Published:** 2021-12-17

**Authors:** Katharina T. Paul, Anna Janny, Katharina Riesinger

**Affiliations:** Department of Political Science, Faculty of Social Sciences, University of Vienna, 1010 Vienna, Austria; anna.janny@univie.ac.at (A.J.); katharina.riesinger@univie.ac.at (K.R.)

**Keywords:** digitalization, health, data, policy, vaccination, immunization, Austria, COVID-19

## Abstract

In this study, we explore the recent setup of a digital vaccination record in Austria. Working from a social-scientific perspective, we find that the introduction of the electronic vaccination pass was substantially accelerated by the COVID-19 pandemic. Our interviews with key stakeholders (*n* = 16) indicated that three main factors drove this acceleration. The pandemic (1) sidelined historical conflicts regarding data ownership and invoked a shared sense of the value of data, (2) accentuated the need for enhanced administrative efficiency in an institutionally fragmented system, and (3) helped invoke the national vaccination registry as an indispensable infrastructure for public health governance with the potential to innovate its healthcare system in the long term.

## 1. Introduction

Since the 1980s, our world has seen an enormous increase in information flow. The advent of personal computers and the World Wide Web has brought with it a wave of digitization, including an increasing amount of data collection and data storage through digital means, due to technological advancements [1]. This process, the “transformation of social action into online (digital) quantified data”, is called datafication [2]. The increased datafication and digitalization of our world concerns almost all areas of life and has been particularly transformational for health governance. This paper addresses one such transformational, but contested, instance: the digitalization of vaccination governance in Austria. We ask: *how was the electronic vaccination record designed and implemented in Austria, and with what implications for governance?*

Institutions providing healthcare attribute “high potential for improving the efficiency and quality of the provided services” [3] to evolving eHealth systems and services including, but not limited to: electronic health records; online information platforms; implanted digital devices; digital imaging systems; and digital disease and immunization registries. Although digitalization in vaccination governance remains under-researched, this area can particularly benefit from it, as the recent success of pandemic policies in Denmark and Israel suggest. In these countries, digital registries were integral in reaching residents and achieving a high vaccination rate. While the importance of epidemiological data availability has become clear during the COVID-19 pandemic and has, in many countries, served as a basis of justification for crisis management measures [4], calls for easily, internationally comparable vaccination information and measurable outcomes of health policies and vaccination campaigns predate the pandemic. In 2012, well before the first COVID-19 outbreaks, the European Commission called for increased interoperability of vaccination information systems and emphasized the need for internationally comparable immunization data [5]. Additionally, in 2018, the EU Commission and the Council of the European Union called on their member states to establish and advance digitalized, centralized vaccination registries, facilitating the international exchange of data [6].

The reliance on datafication in immunization policy has also been met with critical review. Fisher [7] argues that vaccination coverage rates as central indicators can be impenetrable and intransparent for anyone but the institutions producing them (namely the World Health Organization and UNICEF), since these indicators are perceived “as scientific, technical, requiring expertise” [7]; competencies which even the concerned states themselves (supposedly) lack [7]. Similarly, digital health practices and eHealth strategies in the policy field have somewhat shifted the focus of the debate from the political to the technical [8], whereby metrics are framed as neutral and lend objectivity and legitimacy to policy decisions [7,9]. Yet, as Porter has shown, “technologies of data collection are inherently social and political” [10,11]; the production and use of (health) data are by no means “neutral” or “unbiased” and rather need to be seen in the context of the systems (political, economic, societal) which shape them and which, in turn, are shaped by them. These emerging technologies raise questions regarding the responsibility and accountability of the individual and the state towards each other and society at large, regarding the (selective) use of digital data to “enhance and monitor performance” [12]. Data then becomes a form of (social, political, economic) capital that embodies values and carries normative importance [13].

This paper explores a topical and ongoing instance of digitalization in public health, the introduction of a digital vaccination record (called *e-Impfpass* in German) and the entry of vaccination data into a national vaccination registry in Austria. These instruments represent the latest step in a long and ongoing eHealth implementation process embedded in a highly fragmented federalist system, bringing together many different institutions and stakeholders with sometimes conflicting views of data and its value. This very recent project was directly influenced and accelerated in its planning and implementation phases by the ongoing pandemic. As such, it offers insights into future implications for datafication and digitization processes in an area of health care that is currently under considerable pressure [14].

## 2. Materials and Methods

This paper rests on desk research of policy documents, ongoing media coverage, and relevant scientific literature building on searches in PubMed and Web of Science. In addition, we conducted interviews with relevant stakeholders from December 2020 to March 2021, during the implementation stage of the Austrian digital vaccination record, the *e-Impfpass*. This was made possible through an informal cooperation with ELGA GmbH, the legal entity responsible for the implementation and administration of the Austrian electronic health record program. Finally, we build on existing research including interviews from a previous project on regional vaccination registries and comparative work conducted by the first author on vaccination policy across countries (see Supplementary Material, Table S1, for a list of interviews) [15]. Interviews (*n* = 16) were analyzed for common themes, key moments in the history of eHealth in Austria, and perceptions of and involvement in the rolling out of the electronic vaccination pass in 2020–2021.

In this paper, we proceed as follows: first, we briefly introduce vaccination registries as instruments of health governance more generally, before describing the historical development of eHealth in the Austrian context. Next, we trace the development of the digital vaccination record (*e-Impfpass*) from its early stages to its implementation, which was accelerated by the outbreak of the COVID-19 pandemic. We then discuss the value of data to different stakeholders, including political and administrative value. We conclude by discussing the potential implications of the unusually rapid introduction of digitalization in this case study.

## 3. Results

### 3.1. Background

#### 3.1.1. What Are Vaccination Registries?

Registries constitute technical infrastructures that record (all or some) administered vaccinations to calculate vaccination rates, and (regional) overviews of immunization coverage, to calculate risk, curb outbreaks and to send reminders to those due for vaccination or booster doses [16]. However, beyond this, registries can also be used to present transparent data both on adverse reactions to vaccinations, as well as on the benefits of vaccinations. In this way, registries can be used to enhance the effectiveness of vaccination programs by promoting public confidence in the safety of vaccines [17] and in the integrity of the underlying infrastructure.

As such, registries are not merely technical tools, but can serve a variety of governance functions. Accordingly, the literature on vaccination registries identifies a considerable diversity of practices and infrastructures across countries [15]. From a political science perspective, these idiosyncrasies and the diversity in monitoring practices must be understood against the background of differing vaccination systems that are shaped by historical developments, different interests, and institutional path dependency [18]. For instance, the digital and centralized nature of immunization registries in Nordic countries and the Netherlands speak to high levels of trust in governmental institutions, and are in line with centralized governance traditions, whereas the fragmentation of registries in Italy, Austria, and Germany tends to emerge from decentralized forms of governance and a tendency to maintain regional competencies.

#### 3.1.2. Context: eHealth & Electronic Health Records in Austria

The newly emerging Austrian national vaccination registry is inseparably linked to longstanding but contested efforts to promote eHealth and digital health applications in Austria, at the heart of which lies ELGA (short for “Elektronische Gesundheitsakte” or electronic health record, EHR). ELGA constitutes both a data infrastructure and an ongoing process of digitization. Understanding its history and earlier applications is a necessary foundation for our analysis.

Gunter and Terry [19] define electronic health records as “a longitudinal collection of electronic health information about individual patients and populations.” The increasing amount of patient data and its exchange between an increasing number of healthcare providers as well as technological advancements, have made it possible and necessary to digitize data and create safe, reliable, and efficient data entry and data access processes accordingly [20,21]. In Austria, committees working towards a national eHealth strategy were installed as early as 1995, but the introduction of a chip card system (“e-Card”) for patient identification and insurance coverage checks, and the according legal provisions were only passed by the Austrian parliament in 2002. Since 2009, a legal entity called ELGA GmbH has been responsible for the eHealth program of the same name. It is funded by the three main Austrian stakeholders in public health: the federal government, the nine state governments, and the Main Association of Austrian Social Security Institutions, in equal parts [20].

ELGA was tasked with the organizational and technical implementation of the “ELGA Masterplan”, which laid out the long-term strategy for the EHR and other eHealth projects in Austria. This policy program has been identified by scholars as one of the key prerequisites for successful EHR adoption, next to a proper legal basis (which, in Austria, includes the possibility to opt out of the program entirely) [22,23,24,25]. In 2012, however, the program came into heated public discourse due to new legislative proposals regarding EHRs and a subsequent campaign led by the Austrian Medical Association that criticized the emerging eHealth infrastructure. The focus of the debate (and a key argument in the Medical Association’s recommendation for patients to opt out) appears to have been concerns around data protection and privacy. Beyond this, focus group data [26] suggests that possible data misuse for *economic* benefit (e.g., by pharmaceutical companies or insurances) was a more prominent concern with patients. These concerns were to become pertinent in the digitalization of vaccination governance, too.

The public discourse regarding possible data misuse subsided, but the critical position of the Austrian Medical Association continued to shape the implementation of eHealth applications in Austria, such as in the introduction of *e-Medikation*. This application was intended to offer a comprehensive overview of all medication prescribed to patients in order to increase patient safety, ease medication administration (e.g., in care facilities) and prevent medical errors. Shortly after the start of *e-Medikation* as a pilot project in 2011, with three pilot regions participating, the Austrian Medical Association formally boycotted the pilot. This discord between different stakeholders in the implementation process led the *e-Medikation* application roll-out to become significantly delayed; originally planned for 2015, full roll-out in medical practices was not completed until the fall of 2019 [23,24].

Plans for additional ELGA applications had already been outlined in the 2012 “ELGA Masterplan”, namely, patient decrees, precautionary power of attorney, statutory medical registries and the electronic vaccination record (*e-Impfpass*). Yet these projects were delayed, according to one interviewee, for political reasons, some of which had to do with party politics and a high turnover rate of ministers of health. However, it is also likely that this was related to the aforementioned concerns around data privacy and data ownership. With apparently hardly any action taken between 2012 and 2017, the digital vaccination record project gained traction again, starting in 2017, after being (politically) supported by two consecutive health ministers (ITV 2). Although the federal state and provinces had already committed themselves to facilitating its deployment in 2017 in an amendment to the *Zielsteuerung-Gesundheit*, a federal law, ELGA GmbH saw too high a risk of “stranded costs” in pushing project development until a proper legal basis was created in 2020 (during the COVID-19 pandemic).

#### 3.1.3. The Austrian e-Impfpass—Digital Vaccination Record: Features and Functions

In our interviews, we found that stakeholders had taken valuable lessons from the difficult *e-Medikation* project: one interviewee shared the opinion that *e-Medikation* had shown healthcare providers that their work could benefit from such eHealth applications and boosted acceptance of ELGA among them (ITV 4). Simultaneously, ELGA representatives learned that proper incentives were needed to increase support by the Austrian Medical Association; namely, ease of use and simple implementation in medical practices, as well as being open to financial negotiations (ITV 5) and incentives. Additionally, the administrative and medical value of the new registry and its digital vaccination record became very clear in early 2020, when the arrival of COVID-19 pandemic led to an unprecedented nationwide lockdown. Therefore, although *e-Medikation* and the *e-Impfpass* [27] were similar eHealth projects, the conditions under which they were piloted (or, in the case of *e-Impfpass*, planned, changed, and rolled out) were vastly different.

**Figure 1:**
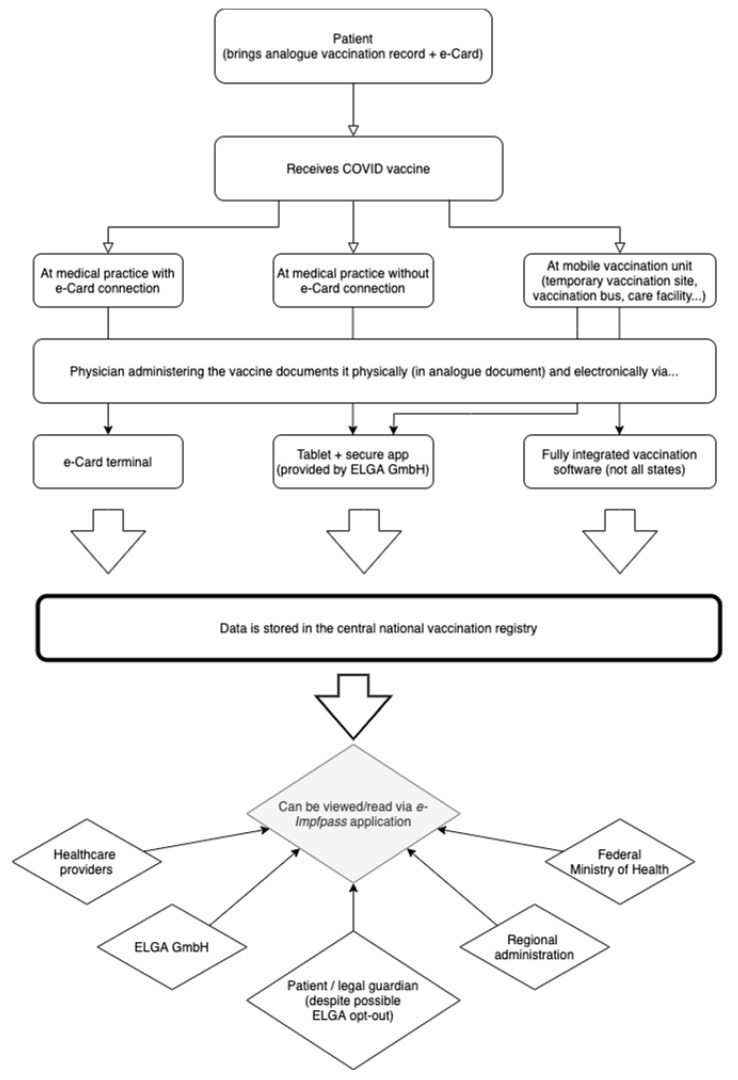
Immunization data governance in Austria.

The Austrian *e-Impfpass* project has had four formalized goals from its inception. First, to supersede paper-based immunization records and eliminate their disadvantages (e.g., accidental loss) by becoming the primary document. The aim for the digital vaccination record to become the new primary immunization document (next to paper-based versions as a secondary document) was, however, always intended for younger cohorts starting with childhood immunization [20], comparable to the Dutch *Præventis* project [16]. There is currently no intention to enter previously administered vaccine doses into the *e-Impfpass*, instead adding only those administered from this point onwards. This might have been one of the reasons why the pilot project was originally focused on childhood immunization. Second, the *e-Impfpass* was to enable the merging of previously fragmented data in a central vaccination registry for increased precision regarding immunization rates and the identification of potential vaccination gaps. Third, the *e-Impfpass* was to help improve crisis management in case of outbreaks and inform targeted and effective measures, especially to help vulnerable population groups. Moreover, the new infrastructure was to simplify the administration of the national immunization program. Finally, the new digital vaccination record allows citizens to access and download their own immunization record, including the EU Digital COVID-19 Certificate [28]. We provide a visualization of these administrative functions in Figure 1 below.

The lead-up to planning and implementing the digital vaccination record was informed by lessons learned from several epidemiological crisis moments, including the H1N1 pandemic in 2009. According to one interviewee (ITV 2), this crisis, along with the introduction of new vaccines such as the vaccine against Human Papilloma Virus (HPV) demonstrated to healthcare administrations the need to have a more precise overview of the number of vaccine doses administered, preferably in a centralized system. Another significant factor were the increased occurrences of measles in Austria, starting with a major outbreak in 2008, which could be traced to an elementary school in Salzburg, spreading throughout Austria to Germany and Norway, with 443 patients infected [29,30]. The digital vaccination record in this context was seen as a crisis management tool to help identify and protect vulnerable persons in case of local outbreaks (ITV 1).

Although the digital vaccination record makes use of extant ELGA IT infrastructure [31], unlike the ELGA program, importantly, the digital vaccination record offers no opt-out possibility for citizens. The option for individuals to opt out of general functions in ELGA is included in the Health Telematics Act (*Gesundheitstelematikgesetz*) (§15 par. 1 GTelG 2012) and has been exercised by 297.000 patients (about 3.4% of all eligible) as of mid-2020 [31]. However, the amended version of the law passed in October 2020 by the Austrian parliament includes legislation specific to the digital vaccination record and the national vaccination registry. It specifies that patients, even those who have previously opted out of the ELGA program, are still obligated to have their COVID-19 immunization data recorded electronically and stored in the national vaccination registry. The law cites “considerable public interest” (using definitions invoked in the EU General Data Protection Regulation) as a justification of not providing individuals with the possibility to opt out (§24b GTelG 2012).

This is not an anomaly; other European countries such as the Netherlands, Sweden and Denmark similarly operate in this fashion, or at least require individual initiative to opt out, for example in recording childhood vaccination [32,33,34,35]. Multiple interview partners in our case study also expressed the imperative of a complete national vaccination registry:

*“That only emerged now during COVID, that we can say, (…) it is mandatory that the vaccine is included in the vaccination registry. For the moment, this only applies to COVID. How it will continue, whether an Opt-Out will be possible in some other way (…) that was definitely a huge point of discussion, which of course accompanied the entire project, and where it came to delays again and again.”*

—ITV 1.

*“And that is, yes and, and, building a national vaccination registry and to say: You can opt out from it, that makes absolutely no sense. But I have also already heard that people don’t want to be vaccinated because of it.”*

—ITV 6.

These interviews reveal the tension between managing the crisis effectively and dealing with longstanding political issues—particularly privacy concerns and individual versus collective responsibility in and for public health. In the immediate crisis that unfolded in 2020, the amended Health Telematics Act was passed comparatively quickly and discussions over data protection and privacy seemed to be overshadowed by the epidemiological emergency narrative. Stakeholders such as the data privacy commission and the chamber of labor criticized this, citing that data privacy concerns by patients as a reason not to get vaccinated against COVID-19 “can hardly be in the public interest” and that, as previous experience has shown, “such centralized registries (…) create potential for misuse” [36]. The rushed legislative process was also criticized by legal experts. The draft bill was highly technical, containing 466 pages of specific technical details which could not be taken into consideration by the data privacy commission during the legal evaluation phase [37]. It is in this sense that the crisis helped to reduce a longstanding political problem to a technical one. The figure below depicts the ways in which data on immunization is collected and governed under the new law.

#### 3.1.4. The Role of the COVID-19 Pandemic

All these factors contributed to the plan of introducing *e-Impfpass* via a pilot project in three Austrian federal states with a focus on childhood vaccination, in close collaboration with pediatricians and, in some cases, school-based doctors administering childhood vaccines. This original pilot project was seen by participating regions as a project that carried prestige, with one province priding itself in its historical role as a trailblazer regarding digital health (ITV5). However, the emergence of the pandemic required quick adaptation and a much faster roll-out than originally planned: rather than childhood vaccinations, recording future vaccinations against COVID-19 in a centralized registry became a top priority epidemiologically, as well as politically.

The data-intensive nature of the pandemic thus served to accelerate the process of digitalization, and what was intended as a pilot project was effectively transformed into a technology roll-out while the technology itself—the *e-Impfpass*—was still in the making. This contingent and ‘unfinished’ nature enabled stakeholders to renegotiate the role and function of the digital vaccination record: the political and epidemiological, if not economic, value of data became apparent and allowed stakeholders, including central and regional authorities as well as professional associations, to jointly push for and support the introduction of the digital vaccination record.

*“[…] and COVID of course, which changed the scope somewhat and added a little pressure, because it probably wouldn’t have called anyone to action whether children between 0 and 6 are included half a year earlier or later, but then, the political pressure was there accordingly and the goals were also pretty clear.”*

—ITV 6.

*“[…] the demand would be in a pandemic to have a real-time vaccination ticker, so to speak, which segment of the population. That’s logical. That is the first thing you know when you start a vaccination campaign: I want to have daily updated numbers so that I can steer things. Also politically. The politicians demand this. And we didn’t have that then.”*

—ITV 2.

Moreover, the sense of urgency, as indicated above, enabled a sidelining of political concerns regarding not only privacy and data protection, but the key question of data ownership—we will return to this below. The pandemic thus meant a reordering of priorities:

*“The pressure is on: we are receiving COVID-vaccines and we will need to document these vaccinations. We have to document them for citizens in a fast way, that’s a matter of patient safety. Because you have two doses, so we have to document the immunization events, we have to document it easily and digitally and we already anticipated, the signals were already very clear during the summer, that the citizens will only be able to go back to their normal lives or do certain things in life only when they can, when we can show that they have been vaccinated.”*

—ITV 5.

The very meaning and (political) function of the digital vaccination record thus shifted: beyond its public health function, digital proof of vaccination was to allow for a return to ‘normal life’. Although this reframing of its function allowed for a faster roll-out—discursively bringing together the collective value of and individual benefit from data collection—it also implied a change in priorities: basic functions such as call-recall systems and reminders were delayed and have not been introduced so far. To sum up, the COVID-19 pandemic provided a unique opportunity for accelerating a long-term project of digitalizing public health in Austria. However, the foregrounding of the need for COVID-19 data implies a backgrounding of basic governance features of the electronic vaccination registry at the cost of improving the national immunization program.

### 3.2. Unpacking the Value of Data

#### 3.2.1. Data and Data Quality as Political Capital

Digitalization processes often touch upon the question of data ownership and use. The conflicting ideas of health data ownership have already been identified as a core conflict in data governance of healthcare and research domains [37,38]. It is thus not surprising that, in the case discussed here, data ownership seems to have different meanings and significance for different stakeholders, including policy officials, healthcare providers, citizens and local health authorities. These issues speak to conceptual understandings of data as a (changing) form of capital [13].

All institutional stakeholders in the Austrian context ascribe political value to vaccination data. The medical, scientific, epidemiological, and societal value of vaccination data seems to be evident to stakeholders, too; yet how this capital may be used, by whom, with which motivation, resources, and competence, varies. For example, for medical doctors, the question of patient (or, in their view, professional) data ownership seems to be important in confirming their “expert” role in the public health system. They also seem to claim, at least partly, ownership over data that they have generated in their professional role. As one interviewee put it: *“you must not forget one thing: a patient file contains the data of many physicians”*—ITV 2. Therefore, vaccination data seems to also present symbolic value and professional capital to them. 

Local authorities, on the other hand, feel ownership over this data because it is collected and aggregated by them, and they would like to be able to use it to answer specific local healthcare matters, likening the data to managerial or organizational capital. Lastly, ELGA see the health data they collect first and foremost as patients’ data, which they help keep safe and provide easier access to for individuals. The question of ownership is necessarily connected to the question of responsibility and accountability, as others have argued [39]. Given the conflicting views of data ownership in the Austrian case, accountability for said data is fragmented: Legally speaking, ELGA are responsible and in charge of the security of all patient data within the ELGA network, including the national vaccination registry, as well as the technical aspects of data entry and IT infrastructure. At the same time, the healthcare providers administering the COVID-19 vaccine are now legally obligated to record this vaccine via the digital vaccination record and are responsible for entering the data correctly. Additionally, regional administrations and newly formed data quality offices are tasked with ensuring data quality and must take accountability politically for vaccination rates in their state. Finally, individuals are called to action by getting vaccinated and checking the data in their files. The fragmented and dispersed accountability for data quality stands in remarkable contrast to earlier efforts for centralization in Austrian vaccination (data) governance. 

These different claims to and responsibilities for immunization data in the national vaccination registry reflect the multiplicity of value understandings of stakeholders regarding vaccination data. Although all stakeholders seem to agree on the medical, epidemiological, and societal capital that vaccination data presents, it seems that each stakeholder group involved in recording, administering and safeguarding vaccination data in turn desires to benefit from the (additional) forms of capital it represents for them. These stakeholder group specific prioritizations lead to differing and sometimes conflicting desires to use data, have access to it (or allow or deny others access) and claim or ascribe responsibility for data quality.

In our interviews, data quality was frequently appealed to as an end in and of itself, but with varying connotations for different stakeholders and actors—not least because of the lack of any national guidelines on what constitutes ‘good data’ or even a consensus on the essential elements of vaccine registries. The digital vaccination record is thus also an attempt at harmonizing these historically grown systems, while at the same time grappling with questions over data ownership and the right to use data for various purposes and with stakeholder-specific intentions. For example, at the moment, local public health departments merely collect data, and feed it into what appears to be somewhat of a “black box” immunization registry. The expectation is that they will receive access to the vaccination data in the future, in order to facilitate their administrative and epidemiological efforts locally. Our interviews indicate that local authorities seek a more active role moving beyond their technical function in vaccination data governance towards a more political role in policy implementation. 

Other stakeholders might have other reasons for wanting access: the scientific community might use data for research purposes, whereas public insurance companies could be interested in improving preventative health measures through a more data-driven approach, including vaccination data. However, as it stands now, the question of future data use by these and other stakeholders seems to be put on hold, at least until after the COVID-19 pandemic has become more manageable; only then, it seems, can further explorations of the value of immunization data in Austria (such a scientific or economic) value for additional stakeholders be discussed.

#### 3.2.2. Administrative Efficiency as a Central Value

Beyond the benefits regarding health policies and the improvement of healthcare systems [16], digital tools also carry administrative value. The logic of administrative efficiency through centralized registries is not unique to the Austrian context; however, it is especially relevant here in that it facilitates the cooperation of different actors in this highly fragmented federalist system through the shared use of vaccination data within an interoperable framework. Epidemiologically, the data is valuable to both states and federal government. States strive to use immunization data most importantly for regional crisis management, and the central government requires this data to report vaccination coverage rates to supranational and intergovernmental agencies and to evaluate vaccination policy. As mentioned above, this is not limited to COVID-19 but also includes, e.g., recurring measles outbreaks.

Reflecting the aims of the registry, an increase in administrative efficiency as well as the ability to draw epidemiological conclusions from data, were key functions for all stakeholders at the stage of project conception and design. In the Austrian context, the aim to “increase administrative efficiency” refers to facilitating the invoicing for vaccination costs between public health insurance providers and Austria’s nine states, especially the fee doctors receive for administering vaccines. Until now, vaccination records are traced and stored differently in each state, which comes with a large administrative effort (and associated cost); therefore, it is in the states’ interest to make this process more efficient. As one interview partner put it, the promise of an increase in administrative efficiency was a key argument when it came to funding the project, highlighting its practical use beyond the pandemic:

*“At some point it became clear, if we introduce a national vaccination registry, if all these vaccination registries that have evolved regionally (…) they all primarily have an accounting purpose. And you will recognize again, also back during the pandemic, the accounting purpose. That means that the implementation of a digital registry is always, I’ll say, very simple and politically clear when it comes to money. If it’s just about the epidemiological aspects, the money doesn’t flow.”*

—ITV 2.

This makes clear that the appeal to administrative efficiency is not merely technical, but political, too, in that it facilitates the move towards centralization in a federalist context, where historically sedimented health care infrastructures—as well as jobs attached to these—hold political and symbolic value. Looking towards the future, it will be crucial to see how the new registry will be expanded beyond the COVID-19 vaccine. As we have demonstrated, the pandemic was an important catalyst for the entire project, driving its development forward at a much faster pace than there would have likely been without it. This, however, may also prove problematic if in the future, it does not gain relevance as a tool for preventative health measures. To do this, the new registry must be expanded to include other vaccinations and become, as was originally planned in the piloting phase, a fixture in the Austrian childhood immunization program. The addition of recall and reminder systems could also be highly beneficial, as research has shown them to be effective in improving immunization rates [39,40].

## 4. Discussion

Our findings demonstrate the impact of the COVID-19 pandemic on digitalization in Austria. In particular, the pandemic helped accelerate the development and implementation of the digital vaccination record and the national vaccination registry in Austria. Moreover, our research supports the idea laid forth by previous scholarly work that the production, collection and use of health data is far from neutral or objective [41,42], but rather an ongoing negotiation and discursive process between the individual and the state [36,39].

First, claims to data ownership and the value ascribed to data are linked, and will invariably differ between multiple stakeholders within the same system, echoing Sadowski’s findings of “data as a core component of political economy in the 21st century” [13]. Second, this framework also ties into the value of administrative efficiency, since according to Sadowski, “data is used to optimize systems” [13]. In the context of a historically grown, fragmented federalist administrative system, combining state specific as well as federal competences, this emerged as a surprisingly important motivation in the design of the digital vaccination record for several stakeholders, suggesting that this data infrastructure, indeed, may serve as a technical solution to a political problem [8]. Third, as we have already mentioned, the national vaccination registry as a data infrastructure serves multiple purposes. As an interoperable platform for administrative purposes (as mentioned above), this also makes immunization data comparable, both nationally and internationally, which is one of the main advantages of eHealth solutions. This also pertains to the idea put forth by Fisher [7] of immunization data being used as a proxy indicator—in this case not to indicate the overall performance of a healthcare system, but rather to indicate the efficiency and success of local and national governments in curbing the COVID-19 pandemic within their realm of influence.

An important aspect of the Austrian vaccination registry, specifically in the context of the pandemic, is its (intended) use regarding the “opening” and access to public life. On the one hand, vaccination cover rates for COVID-19, both on a national and a state-based level, are becoming the second most important metric (after COVID-19 incidence rates) upon which policy decisions are based, or at least with which they are justified. On the other hand, the “Green Pass”, which was introduced nationally in May of 2021, and on a European (interoperable) level as the “EU Digital COVID Certificate” in July 2021, grants increased access to public life (restaurants, cultural events, etc.) to persons who have very recently tested negative for COVID-19, to those harboring antibodies after a previous infection, or to individuals already fully vaccinated.

Both uses of the registry as the basis for COVID-related policies seem to foreshadow increased future use of the digital vaccination record as an important tool of governance for the entirety of Austrian society, as well as on an individual level. Thus, it can be argued that the digital vaccination record as a data infrastructure has, since its inception, gained tremendous meaning in terms of serving metrics (perceived as “neutral” and “unbiased”) to be used as the basis for policy decisions, which then, in turn, once again influence said metrics and the way data is produced [10,39].

It took the COVID-19 pandemic as a cataclysmic event to accelerate the development of this project and without the pandemic, the roll-out and acceptance would probably have taken longer, and a much broader debate over data privacy concerns and the obligatory nature of the program might have ensued. However, the limited public consultation on—rather than only expert discussions on privacy, data ownership and non-governmental use of data, and the uncertain expansion of data collection and use in the future—may come with political costs and may backfire in the long run.

As we look toward the future of the Austrian digital vaccination record, its effectiveness as a true immunization information system (including more vaccines and a broader range of functionalities) remains to be seen. If it is expanded and fully integrated into the existing healthcare infrastructure, it could serve as an important tool for preventive health measures and crisis management. This might improve vaccination rates, especially if recall and reminder systems are incorporated, and serve as a true primary vaccination document, which is internationally compatible, possibly even driving further digitalization efforts in Austrian public healthcare. However, a lack of public discourse around the national vaccination registry, and the value and “ownership” concepts of the data within it, as well as future opt-out possibilities, might generate resistance to vaccination [43] and affect trust in healthcare institutions more generally. 

## Data Availability

Data supporting the reported results are not openly available in order to protect the identity of respondents. Selected transcripts can be made available upon request and contingent upon the consent of the respondent.

## Supplementary Materials

The following are available online at https://www.mdpi.com/article/10.3390/vaccines9121495/s1, Table S1: List of interviews.
