# Supplementary material for: Austria’s Digital Vaccination Registry: Stakeholder Views and Implications for Governance
Source: Vaccines (Basel). 2021 Dec 17;9(12):1495. doi: 10.3390/vaccines9121495 (PMC8706289; doi:10.3390/vaccines9121495)
Supplement: Supplementary file 1 [file vaccines-09-01495-s001.zip › vaccines-1423463-supplementary.pdf]

Table S1. List of interviews.

ITV no.	Date (dd/mm/yy)	Information on interviewee
1	28.10.20	Senior Policy Official
2	14.01.21	Senior Policy Official
3	02.02.21	IT Expert
4	08.02.21	Senior Policy Official
5	17.02.21	Senior Policy Official
6	18.02.21	IT and EHR Expert
7	17.03.21	Senior Medical Official
8	2018	Senior Public Health Official, federal state 1 (W)
9	2018	Senior Public Health Official, federal state 2 (S)
10	2018	Senior Public Health Official, federal state 3 (N)
11	2018	Data Administrator, federal state (N)
12	21.02.18	Senior Immunologist, Medical University
13	06.03.18	Senior Epidemiologist
14	23.05.18	Senior Public Health Official, federal state 4
15	24.05.18	Senior Statistician
16	30.01.19	Senior Statistician (academic)
